# Molecular basis for the functions of dominantly active Y35N and inactive D60K Rheb mutants in mTORC1 signaling

**DOI:** 10.1093/jmcb/mjaa025

**Published:** 2020-05-29

**Authors:** Chunxiao Zhang, Yan Liu, Yifang Zhang, Xiangxiang Wang, Tianlong Zhang, Jianping Ding

**Affiliations:** 1 State Key Laboratory of Molecular Biology, Shanghai Institute of Biochemistry and Cell Biology, Center for Excellence in Molecular Cell Science, University of Chinese Academy of Sciences, Chinese Academy of Sciences, Shanghai 200031, China; 2 School of Life Science and Technology, ShanghaiTech University, Shanghai 201210, China; 3 School of Life Sciences, Shanghai University, Shanghai 200444, China; 4 School of Life Science, Hangzhou Institute for Advanced Study, University of Chinese Academy of Sciences, Hangzhou 310024, China

**Dear** **Editor**,

Mammalian target of rapamycin complex 1 (mTORC1) serves as a central regulator of cell growth and proliferation by integrating signals from growth factors, nutrients, energy status, and cellular stress (Saxton and Sabatini, 2017). A small GTPase, called Ras homolog enriched in brain (Rheb), is a positive regulator of mTORC1. Like other small GTPases, the function of Rheb is dictated by its guanine nucleotide binding states: it is active in the GTP-bound form and inactive in the GDP-bound form (Aspuria and Tamanoi, 2004). Crystal structures of Rheb in complexes with GDP, GTP, and GppNHp (a nonhydrolysable GTP analog) revealed that major conformational change takes place in the switch I region during the GTP‒GDP transition (Yu et al., 2005). Cryo-EM structure of mTORC1 in complex with GTP-bound Rheb suggested an allosteric mechanism for mTORC1 activation by Rheb (Yang et al., 2017). Intriguingly, the interaction of Rheb with mTOR is relatively weak and in a nucleotide-independent manner, which is different from the interactions of classical Ras proteins with their effectors (Long et al., 2005). Nevertheless, the functional data showed that only the GTP-bound Rheb can activate mTOR (Yang et al., 2017). So far, multiple Rheb mutants have been identified (Heard et al., 2014). Among them, the Y35A mutant exhibited increased intrinsic GTPase activity and its overexpression reduced the activation of mTORC1 by growth factor availability, and thus is deemed as a loss-of-function mutant (Mazhab-Jafari et al., 2012; Heard et al., 2014). Surprisingly, the Y35N mutant, which was initially identified in several human cancers (Lawrence et al., 2014), could significantly increase the phosphorylation level of mTORC1 substrate S6K1 compared to the wild-type (WT) Rheb, and thus is regarded as a constitutively active mutant (Grabiner et al., 2014; Heard et al., 2014). On the other hand, the D60K mutant was shown to be unable to bind to Mg^2+^ or the nucleotide (either GTP or GDP) and hence to assume a nucleotide-free form; thus, it is regarded as a dominantly inactive mutant and widely used as a negative control in the functional study of mTORC1 activation (Tabancay et al., 2003). However, how the Y35N and D60K mutations alter the proper function of Rheb in the activation of mTORC1 signaling remains unclear.

In this study, we firstly analyzed the activating effects of WT Rheb and the Y35N and D60K mutants on mTORC1. Our *in vitro* kinase activity assays of mTORC1 show that the GppNHp-bound WT Rheb (designated as WT^GppNHp^) increases the phosphorylation of S6K1, suggesting that it can activate mTORC1, whereas the GDP-bound WT Rheb (designated as WT^GDP^) cannot (Figure 1A and B). These results are in agreement with the previous results (Yang et al., 2017). Surprisingly, both the GppNHp-bound and GDP-bound Y35N mutants (designated as Y35N^GppNHp^ and Y35N^GDP^, respectively) exhibit similar activating ability for mTORC1 as WT^GppNHp^ (Figure 1A). In contrast, the dominantly inactive D60K mutant cannot activate mTORC1 in the presence of either GppNHp or GDP (Figure 1B).

**Figure 1:**
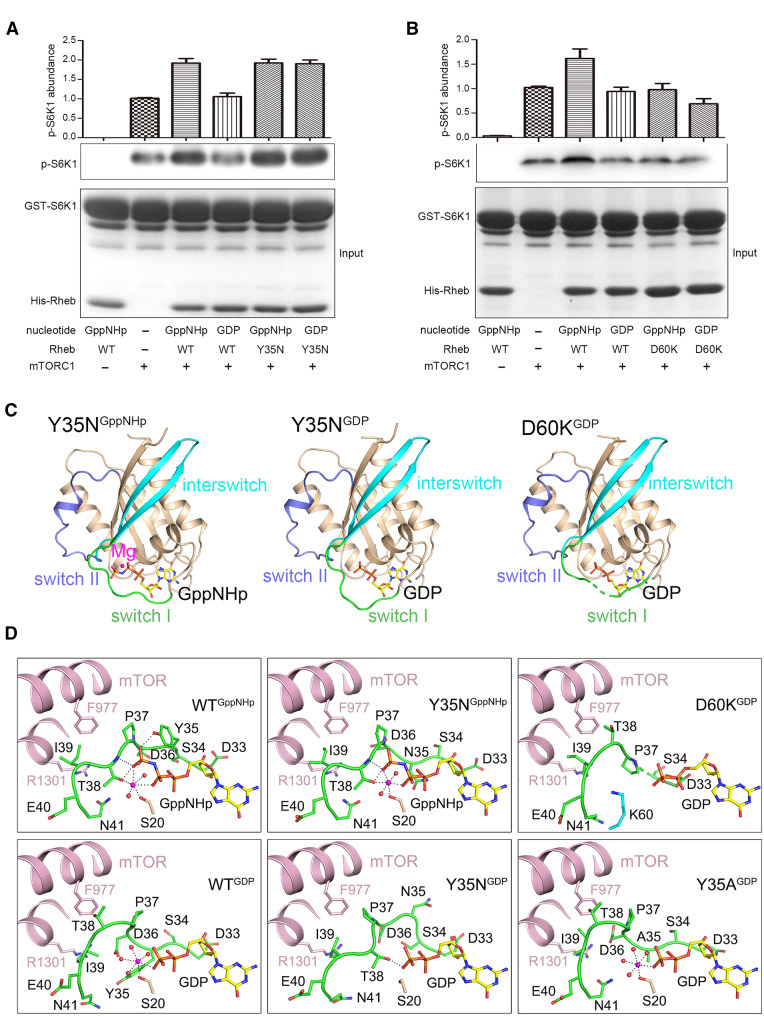
Crystal structures and biochemical characterization of Rheb Y35N and D60K mutants. (**A** and **B**) *In vitro* kinase activity assays of mTORC1 toward S6K1 phosphorylation in the absence or presence of Rheb WT, Y35N (**A**), or D60K (**B**). Band intensities for p-S6K1 are quantified and presented as graphs in the upper panel. The gels for the inputs were stained with Coomassie blue. (**C**) Overall structures of Y35N^GppNHp^, Y35N^GDP^, and D60K^GDP^. The switch I region is colored in green, the switch II region in blue, the interswitch region in cyan, and the other part of Rheb in wheat. Mg^2+^ is shown as a sphere in magenta, and the nucleotide with a ball-and-stick model in yellow. (**D**) Interactions between the switch I regions of WT and mutant Rheb with mTOR. The structure models are constructed by superimposing the structures of WT^GppNHp^ (PDB code 1XTR), WT^GDP^ (PDB code 1XTQ), Y35N^GppNHp^, Y35N^GDP^, D60K^GDP^, and Y35A^GDP^ (PDB code 3SEA) onto the structure of WT Rheb in the cryo-EM structure of Rheb‒mTORC1 complex (PDB code 6BCU). The residues involved in the interactions are shown with ball-and-stick models and colored as in **C.**

To investigate the molecular basis for the constitutive activation of mTORC1 by the Y35N mutant, we determined the crystal structures of Y35N^GppNHp^ and Y35N^GDP^ at 2.0 Å and 2.1 Å resolution, respectively (Figure 1C;Supplementary Table S1). The Y35N mutant assumes a very similar overall structure as the WT Rheb with a typical small GTPase protein fold composed of a six-stranded β-sheet surrounded by four α-helices (Figure 1B). The overall structure of Y35N^GppNHp^ is very similar to that of WT^GppNHp^; superposition of the two structures using the secondary-structure matching method yields a root mean square deviation (RMSD) of 0.45 Å for 169 Cα atoms. On the other hand, the overall structure of Y35N^GDP^ is more similar to that of WT^GppNHp^ (RMSD of 0.79 Å for 162 Cα atoms) than that of WT^GDP^ (RMSD of 0.92 Å for 162 Cα atoms) or Y35A^GDP^ (RMSD of 0.97 Å for 167 Cα atoms). In both structures, the switch I (residues 33‒41), interswitch (residues 42‒62), and switch II (residues 63‒79) regions of Rheb and the bound nucleotide are well defined in the electron density map (Supplementary Figures S1A, B and S2A, B). In the Y35N^GppNHp^ structure, there is a Mg^2+^ bound at the active site, which is coordinated by the side chains of Ser20 and Thr38, the β-phosphate and γ-phosphate of GppNHp, and two water molecules in an octahedral geometry. In the Y35N^GDP^ structure, no Mg^2+^ could be identified at the active site, although Mg^2+^ is present in the protein storage buffer and the crystallization solution (Figure 1B and C).

In both of the Y35N^GppNHp^ and Y35N^GDP^ structures, the Y35N mutation renders the switch I region to assume conformations different from those in the WT and Y35A structures, whereas the switch II and interswitch regions could be overlapped very well (Figure 1D;Supplementary Figure S3A and B). In WT^GppNHp^, the side chain of Tyr35 covers on the top of the phosphate-binding pocket and forms a hydrogen bond with the γ-phosphate of GppNHp. In Y35N^GppNHp^, the side chain of Asn35 flips into the bottom of the phosphate-binding pocket and points toward the α-phosphate and β-phosphate of GppNHp (about 90° rotation relative to that of Tyr35 in WT^GppNHp^). Besides, the binding modes of GppNHp and Mg^2+^ are essentially identical to those in WT^GppNHp^. In particular, Thr38 makes a coordination bond with Mg^2+^ via its side chain and forms hydrogen bonds with the γ-phosphate of GppNHp via its main chain and side chain. In contrast, the switch I region and the binding mode of GDP exhibit notable difference in Y35N^GDP^ and WT^GDP^ (RMSD of 2.2 Å for 9 Cα atoms of the switch I region using the least-squares fitting method). In WT^GDP^, Mg^2+^ is coordinated by the side chain of Ser20, the β-phosphate of GDP, and four water molecules. Compared to that in WT^GppNHp^, the side chain of Tyr35 flips into the bottom of the phosphate-binding pocket and points toward the Mg^2+^-binding site and the side chain of Thr38 flips out of the active site and points toward the solvent. Similarly, in Y35A^GDP^, the switch I region adopts a similar conformation as that in WT^GDP^, suggesting that mutation Y35A does not affect the conformation of switch I. However, in Y35N^GDP^, the side chain of Asn35 flips away from the nucleotide and points toward the solvent (about 180° rotation from that of Asn35 in Y35N^GppNHp^). Meanwhile, the side chain of Thr38 extends slightly closer toward the nucleotide, and thus occupies in part the position of Mg^2+^ and forms a hydrogen bond with the β-phosphate of GDP. These results indicate that in both GDP- and GTP-bound Y35N structures, the switch I region and in particular the side chain of Asn35 assume unique conformations different from those of WT, leading the side chain of Thr38 to adopt a conformation similar to that in WT^GppNHp^ (Figure 1D;Supplementary Figure S3C). These conformational differences are very likely attributed to the Y35N mutation.

In the Rheb^GTP^‒mTORC1 structure, switch I of Rheb interacts only with the M-HEAT and FAT domains of mTOR, while switch II interacts with all the N-HEAT, M-HEAT, and FAT domains, bringing them into the proper positions for catalysis (Yang et al., 2017). When the WT and Y35N Rheb structures are superimposed onto the Rheb‒mTORC1 structure, residues Pro37 and Ile39 of switch I in both WT^GppNHp^ and Y35N^GppNHp^ could interact with Phe977 and Arg1301 of mTOR (Figure 1D). When the WT^GDP^ structure is superimposed onto the Rheb‒mTORC1 structure, due to the conformational changes of switch I and in particular Tyr35 and Thr38, the side chain of Thr38 would have a steric clash with Phe977 of mTOR. These results indicate that the conformations of Tyr35 and Thr38 of Rheb play a vital role in its interaction with mTOR and thus the activation of mTORC1. In the Y35N^GDP^ structure, the switch I region assumes a similar conformation as WT^GppNHp^ and Y35N^GppNHp^ and, in particular, the side chain of Thr38 is oriented toward the nucleotide to avoid steric conflict with mTOR, and hence both Pro37 and Ile39 are in proper positions to make tight interactions with mTOR. In other words, Y35N^GDP^ mimics WT^GppNHP^ in binding to mTOR and thus is capable to activate mTORC1 in a similar way as WT^GppNHp^. These results indicate that the Y35N mutation induces switch I in either GDP-bound or GppNHp-bound form to adopt a conformation similar to that seen in WT^GTP^ and thus can make a tight interaction with mTOR, leading to constitutive activation of mTORC1. This provides the structural basis for why the Y35N mutant can activate mTORC1 in both GppNHp- and GDP-bound forms (Figure 1A) and functions as a constitutively active mutant in mTORC1 signaling, which causes the hyper activation of mTORC1 and promotes tumor growth.

To investigate the molecular basis for the incapability of the D60K mutant to activate mTORC1, we determined the crystal structure of D60K^GDP^ at 2.6 Å resolution (Figure 1C;Supplementary Table S1). Surprisingly, there is a GDP bound at the active site of the D60K mutant with clearly defined electron density, even though no GDP was added in the protein purification and crystallization processes (Supplementary Figures S1C and S2C), which is in disagreement with the previous functional data showing that the D60K mutant assumes a nucleotide-free form. Nevertheless, consistent with the previous functional data, there is no Mg^2+^ bound at the active site (Tabancay et al., 2003; Figure 1C and D). Intriguingly, attempts to co-crystallize the D60K mutant with GppNHp failed to yield any crystals. In the D60K^GDP^ structure, the long, positively charged side chain of Lys60 is extended into the Mg^2+^-binding site, which not only hinders the Mg^2+^ binding owing to electrostatic repulsion but also expels the side chain of Thr38 away from the active site, to adopt a conformation similar to that seen in WT^GDP^ but different from that seen in Y35N^GDP^ (Figure 1D;Supplementary Figure S4). In addition, distinct from all known WT and mutant Rheb structures, in the D60K^GDP^ structure, a portion of switch I (residues 35 and 36) is disordered. It is very likely that the lack of Mg^2+^ at the active site makes the binding of the negatively charged nucleotides (GDP and in particular GTP) less stable and increases the flexibility of switch I. These results indicate that the D60K mutation renders the Rheb mutant unable to bind to Mg^2+^, which leads to not only the incapability of nucleotide binding but also the partial disordering of switch I and the side chain of Thr38 to adopt a conformation similar to that seen in WT^GDP^. This explains why the D60K mutant cannot activate mTORC1 (Figure 1B) and functions as a constitutively inactive mutant in mTORC1 signaling.

*[Supplementary material is available at Journal of Molecular Cell Biology online. The crystal structures of the Y35N^GppNHp^, Y35N^GDP^, and D60K^GDP^ mutants have been deposited with the RCSB Protein Data Bank (accession code 7BTD, 7BTC, and 7BTA). We thank the staff at BL17U1 of Shanghai Synchrotron Radiation Facility (SSRF) and BL18U and BL19U1 of National Facility for Protein Science in Shanghai (NFPSS) for technical assistance in diffraction data collection. This research was supported by grants from Chinese Academy of Sciences (XDB37030305) and the National Natural Science Foundation of China (31530013 and 31870722).]*

## Supplementary Material

mjaa025_Supplementary_material
