# Supplementary material for: Molecular basis for the functions of dominantly active Y35N and inactive D60K Rheb mutants in mTORC1 signaling
Source: J Mol Cell Biol. 2020 May 29;12(9):741–4. doi: 10.1093/jmcb/mjaa025 (PMC7749741; doi:10.1093/jmcb/mjaa025)
Supplement: mjaa025_Supplementary_material [file mjaa025_supplementary_material.pdf]

Supplementary material

Molecular basis for the functions of dominantly active Y35N and inactive D60K Rheb mutants in mTORC1 signaling

Chunxiao Zhang[1], Yan Liu[2], Yifang Zhang[1], Xiangxiang Wang[3], Tianlong Zhang[1,*], and Jianping Ding[1,*]

[1] State Key Laboratory of Molecular Biology, Shanghai Institute of Biochemistry and Cell Biology, Center for Excellence in Molecular Cell Science, University of Chinese Academy of Sciences, Chinese Academy of Sciences, Shanghai 200031, China

[2] School of Life Science and Technology, ShanghaiTech University, Shanghai 201210, China

[3] School of Life Sciences, Shanghai University, Shanghai 200444, China

* Correspondence to: Tianlong Zhang, Tel: +86-21-5492-1115, E-mail: tlzhang@sibcb.ac.cn; Jianping Ding, Tel: +86-21-5492-1619, E-mail: jpding@sibcb.ac.cn

Running title: Crystal structures of Y35N and D60K Rheb mutants

Keywords: mTORC1 signaling, Rheb, small GTPase, crystal structure

Supplementary Materials and methods

Cloning, expression and purification of proteins

The Y35N and D60K Rheb mutants were generated using QuikChange Site-Directed Mutagenesis kit (Stratagene). The wild-type Rheb in the pET-22b(+)-His vector (Novagen) was used as the template (Yu et al., 2005). The mutations were verified by sequencing (Sangon). The wild-type Rheb and mutants were expressed and purified as described previously (Yu et al., 2005). Cells were harvested by centrifugation, resuspended in a lysis buffer (30 mM Tris-HCl, pH 7.5, 1 mM $MgCl_2$, and 150 mM NaCl), and lysed by sonication. Protein purification was carried out by affinity chromatography using an Ni-NTA column (Qiagen) with the lysis buffer supplemented with 20 mM and 200 mM imidazole serving as washing buffer and elution buffer, respectively. To obtain homogeneous GppNHp-bound and GDP-bound Y35N, the purified Y35N mutant protein was exchanged into a buffer (10 mM Hepes, pH 7.5, 5 mM EDTA, 150 mM NaCl, and 1 mM DTT) in an Amicon ultrafiltration tube (Pharmacia) and then incubated for 1 hr to remove the bound metal ion and nucleotide. The yielded protein solution was further exchanged to the storage buffer (10 mM Hepes, pH 7.5, 1 mM $MgCl_2$, 150 mM NaCl, and 1 mM DTT) and then supplemented with 10 mM $MgCl_2$ and 10-fold excess of GppNHp or GDP. On the other hand, the purified D60K mutant protein was simply exchanged to the storage buffer without addition of any nucleotides. The mutant proteins were finally purified by gel filtration using a Superdex 200 10/60 column (GE Healthcare) pre-equilibrated with the storage buffer for structural and biochemical studies.

The GST-fused human $S6K1^{367-404}$ protein was expressed in *E. coli* using the pET-M30 vector. Protein was purified by glutathione affinity chromatography and dialyzed into a buffer (30 mM Tris-HCl, pH 7.5, 200 mM NaCl, 2 mM DTT, and 10% glycerol). The protein was

concentrated to ~3 mg/mL (100 μM) and stored in -80 ℃ after aliquoted.

For the expression of human mTORC1 complex, full-length mTOR, Raptor, and mLST8 were individually sub-cloned into the modified pCAG vector which was kindly provided by Dr. Yanhui Xu (Fudan University, Shanghai, China) (Yang et al., 2016). The three plasmids were co-transfected to HEK293T cells by using Lipofectamine 2000 (Invitrogen) according to the manufacturer's instructions. Cells were cultured in Dulbecco's modified Eagle's medium supplemented with 10% fetal bovine serum (FBS) at 37 ℃ in a 5% CO_2 incubator for 48 hr. To make serum and amino acid starvation, the cells expressed mTORC1 were washed twice with Dulbecco's Phosphate-Buffered Saline (D-PBS, Invitrogen) and incubated in D-PBS for 1 hr (Sato et al., 2008). The cells were collected and lysed in the lysis buffer (50 mM Tris-HCl, pH 7.5, 150 mM NaCl, 10 mM $MgCl_2$, 10% glycerol, 20 mM β-glycerophosphate, 0.4% CHAPS, 1 mM PMSF) at 4 ℃ for 1h. Protein was harvested using anti-DYKDDDDK G1 affinity resin (GenScript) and eluted with the lysis buffer supplemented with flag peptide.

In vitro kinase activity assay

In vitro kinase activity assay of mTORC1 was performed in the reaction buffer containing 25 mM Hepes, pH 7.4, 100 mM NaCl, 10 mM $MgCl_2$, 5% glycerol, and 2 mM DTT. Reactants were assembled and incubated on ice for 10 min. The reaction was initiated with the addition of ATP at the final concentration of 0.5 mM at 37 ℃ and after 30 min, the reaction was terminated with the addition of SDS loading buffer. The reaction mixture was boiled at 95 ℃ and then analyzed by SDS-PAGE and immunoblotting with antibody against pThr389 S6K1 (Cell Signaling Technology). Band intensities for p-S6K1 were quantified by ImageJ (Schneider et al., 2012).

Crystallization, data collection and structure determination

Crystallization was carried out at 20 ℃ using the hanging drop vapor diffusion method by mixing equal volumes of protein solution (about 10 mg/mL) and reservoir solution. Crystals of Y35N^{GppNHp} were grown in drops containing the reservoir solution of 0.2 M ammonium acetate, 0.1 M Hepes, pH 7.5, 25% (w/v) PEG 3350. Crystals of Y35N^{GDP} were grown from drops containing the reservoir solution of 0.1% n-octyl-β-D-glucoside, 0.1 M sodium citrate, pH 5.5, and 22% (w/v) PEG 3350. Crystals of D60K^{GDP} were grown from drops containing the reservoir solution of 0.2 M sodium phosphate and 16% (w/v) PEG 3350. The crystals were cryoprotected using the reservoir solution supplemented with 30% glycerol and then flash-cooled in liquid N_2. Diffraction data were collected at -175 ℃ at BL19U1 of National Facility for Protein Science in Shanghai (NFPSS) and BL17U of Shanghai Synchrotron Radiation Facility (SSRF), China, and were processed, integrated, and scaled together with XDS (Kabsch, 2010) or HKL3000 (Minor et al., 2006).

The structures of the Rheb mutants were solved by the molecular replacement (MR) method implemented in Phenix (Adams et al., 2010) using the structures of the wild-type Rheb^{GTP} (PDB code 1XTS) or Rheb^{GDP} (PDB code 1XTQ) as search models. Structure refinement was carried out using Phenix and Refmac5 (Adams et al., 2010; Murshudov et al., 2011). Model building was performed manually using Coot (Emsley and Cowtan, 2004). Structural analysis was carried out using programs in the CCP4 suite (Winn et al., 2011). Structure figures were generated using Pymol (Schrodinger, 2015). Statistics of the structure refinement and the quality of the final structure models are summarized in **Supplementary Table S1**.

The crystals of Y35N^{GppNHp} belong to space group $C222_1$ and contain one Rheb in the asymmetric unit. The structure model of Y35N^{GppNHp} consists of residues 3-171 of Rheb, a GppNHp and a Mg^{2+}. The crystals of Y35N^{GDP} belong to space group $P2_12_12_1$ and contain four

Rheb molecules in the asymmetric unit which exhibit no notable conformational differences. In all the four monomers, there is a GDP bound at the active site, but no Mg^{2+} ion was identified in the electron density map. Monomer A contains more defined residues (residues 2-170) of Rheb with well-defined switch regions and thus is used for structural analysis. The crystals of $D60K^{GDP}$ belong to space group $C2_1$ with two Rheb molecules per asymmetric unit. Monomer A contains residues 4-34 and 37-170 of Rheb and is used for structural analysis.

Supplementary References

Adams, P.D., Afonine, P.V., Bunkoczi, G., et al. (2010). PHENIX: a comprehensive Python-based system for macromolecular structure solution. Acta Crystallogr. *D66*, 213-221.

Emsley, P., and Cowtan, K. (2004). Coot: model-building tools for molecular graphics. Acta Crystallogr. *D60*, 2126-2132.

Kabsch, W. (2010). Xds. Acta Crystallogr. *D66*, 125-132.

Mazhab-Jafari, Mohammad T., Marshall, Christopher B., Ishiyama, N., et al. (2012). An Autoinhibited Noncanonical Mechanism of GTP Hydrolysis by Rheb Maintains mTORC1 Homeostasis. Structure *20*, 1528-1539.

Minor, W., Cymborowski, M., Otwinowski, Z., et al. (2006). HKL-3000: the integration of data reduction and structure solution--from diffraction images to an initial model in minutes. Acta Crystallogr. *D62*, 859-866.

Murshudov, G.N., Skubak, P., Lebedev, A.A., et al. (2011). REFMAC5 for the refinement of macromolecular crystal structures. Acta Crystallogr. *D67*, 355-367.

Sato, T., Umetsu, A., and Tamanoi, F. (2008). Characterization of the Rheb-mTOR signaling pathway in mammalian cells: constitutive active mutants of Rheb and mTOR. Methods Enzymol. *438*, 307-320.

Schneider, C.A., Rasband, W.S., and Eliceiri, K.W. (2012). NIH Image to ImageJ: 25 years of image analysis. Nat. Methods *9*, 671-675.

Schrodinger, LLC. (2015). The PyMOL Molecular Graphics System, Version 2.0 Schrödinger, LLC.

Winn, M.D., Ballard, C.C., Cowtan, K.D., et al. (2011). Overview of the CCP4 suite and current developments. Acta Crystallogr. *D67*, 235-242.

Yang, H., Wang, J., Liu, M., et al. (2016). 4.4 A Resolution Cryo-EM structure of human mTOR Complex 1. Protein & cell *7*, 878-887.

Yu, Y., Li, S., Xu, X., et al. (2005). Structural basis for the unique biological function of small GTPase RHEB. J. Biol. Chem. *280*, 17093-17100.

Supplementary Figures

A

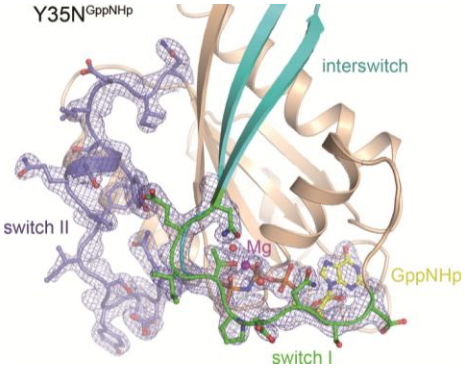

B

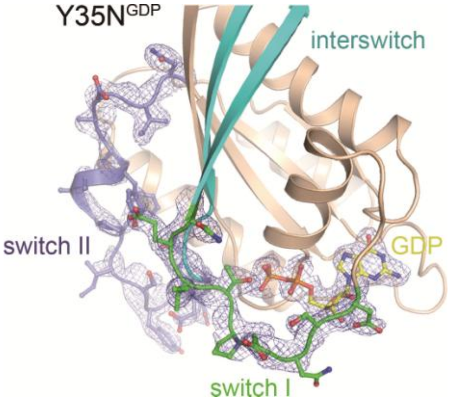

C

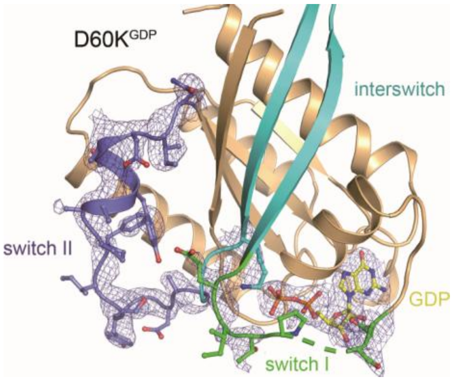

Supplementary Figure S1 Composite simulated annealing 2Fo-Fc omit map (contoured at 1σ) for the swich regions of Y35N^{GppNHp} (**A**), Y35N^{GDP} (**B**), and D60K^{GDP} (**C**). The structure elements are colored as in Figure 1C.

A

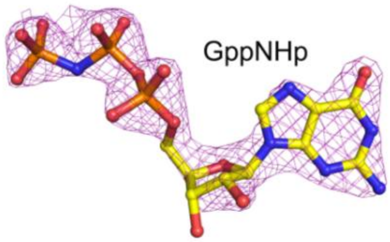

B

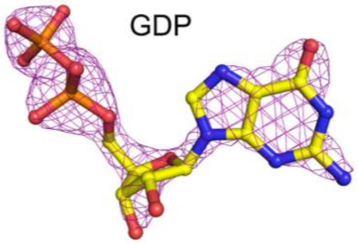

C

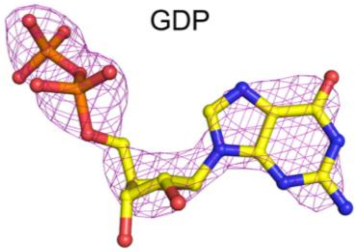

Supplementary Figure S2 Composite simulated annealing Fo-Fc omit map (contoured at 3σ) for the bound nucleotide in Y35N^{GppNHp} (**A**), Y35N^{GDP} (**B**), and D60K^{GDP} (**C**).

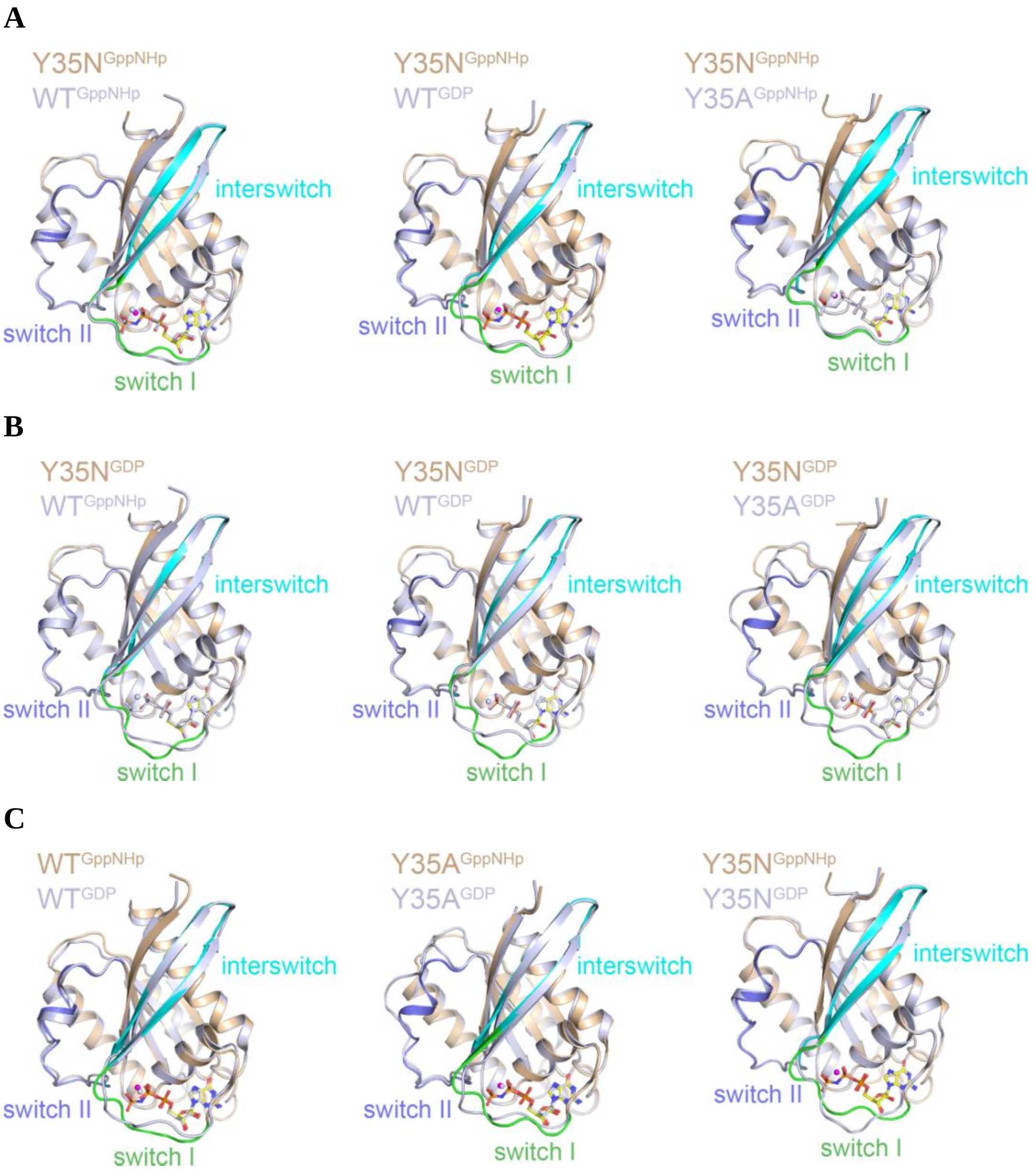

Supplementary Figure S3 Comparison of different Rheb structures. (**A**) Superposition of $Y35N^{GppNHp}$ with WT^{GppNHp} (PDB code 1XTR), WT^{GDP} (PDB code 1XTQ), and $Y35A^{GppNHp}$ (PDB code 3SEA) (Mazhab-Jafari et al., 2012). (B) Superposition of $Y35N^{GDP}$ with WT^{GppNHp} (PDB code 1XTR), WT^{GDP} (PDB code 1XTQ), and $Y35A^{GDP}$ (PDB code 3SEA). (C) Superposition of GppNHp-bound or GDP-bound WT (PDB code 1XTR and 1XTQ), Y35A (PDB code 3SEA), and Y35N.

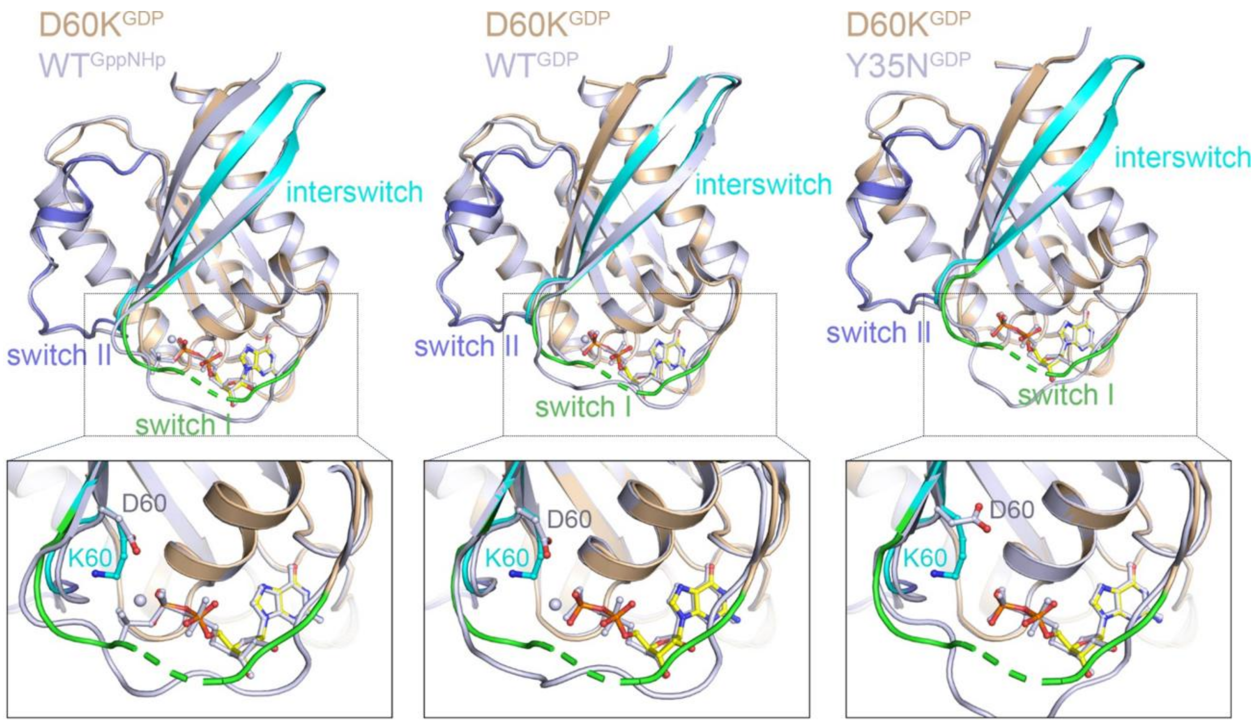

Supplementary Figure S4 Superposition of D60K^{GppNHp} with WT^{GppNHp} (PDB code 1XTR), WT^{GDP}(PDB code 1XTQ), and Y35N^{GDP}. Zoom-in view of the side chain of Lys60 shows the steric clash with the Mg^{2+} binding.

Supplemental Table S1 Statistics of X-ray diffraction data and structure refinement.

	**D60K^{GDP}**	**Y35N^{GDP}**	**Y35N^{GppNHp}**
**Diffraction data**			
Wavelength (Å)	0.9792	0.9778	0.9785
Space group	$C2_1$	$P2_12_12_1$	$C222_1$
Cell parameters			
a (Å)	108.9	75.4	97.6
b (Å)	47.8	78.7	103.0
c (Å)	61.1	117.4	47.7
β (°)	112.7	90.0	90.0
Resolution (Å)	31.80-2.60 (2.69-2.60)[a]	39.30-2.10 (2.18-2.10)	50.00-2.00 (2.07-2.00)
Observed reflections	37,057	524,442	118,186
Unique reflections ($I/\sigma(I) > 0$)	9,007	41,416	16,650
Average redundancy	4.0 (3.2)	12.7 (9.7)	7.1 (6.3)
Average $I/\sigma(I)$	14.1 (2.5)	23.0 (2.4)	19.0 (2.2)
Completeness (%)	97.8 (79.1)	99.9 (97.7)	99.1 (99.8)
R_{merge} (%)[b]	7.4 (59.3)	10.8 (57.2)	10.0 (54.7)
$CC_{1/2}$	0.998 (0.881)	0.999 (0.902)	0.996 (0.900)
**Refinement and structure model**			
Reflections ($Fo \geq 0\sigma(Fo)$)			
Working set	8,540	37,273	15,780
Test set	423	1,991	833
R_{work}/R_{free} (%)[c]	19.1/25.6	18.7/23.3	16.4/20.4
No. of protein atoms	2,411	5,216	1,340
No. of ligand atoms	66	112	32
No. of Mg^{2+} atoms	–	–	1
No. of solvent atoms	12	249	105
Average B factor (Å^2)			
Protein	51.6	45.9	37.6
Ligand	46.6	36.3	30.5
Mg^{2+}	–	–	32.1
Solvent	36.8	43.1	46.5
RMS deviation			
Bond lengths (Å)	0.009	0.007	0.009
Bond angles (°)	1.2	1.2	1.2
Ramachandran plot (%)			
Favored	96.7	97.4	98.2
Allowed	3.3	2.6	1.8
Outliers	0.0	0.0	0.0

[a] Numbers in parentheses refer to the highest resolution shell.

[b] $R_{merge}=\sum_{hkl}\sum_i |I_i(hkl)_i - \langle I(hkl)\rangle| / \sum_{hkl}\sum_i I_i(hkl)$.

[c] R factor$=||F_o|-|F_c||/|F_o|$.
